# Translation and Translational Control in Dinoflagellates

**DOI:** 10.3390/microorganisms6020030

**Published:** 2018-04-07

**Authors:** Sougata Roy, Rosemary Jagus, David Morse

**Affiliations:** 1 Institut de Recherche en Biologie Végétale, Département de Sciences Biologiques, Université de Montréal, 4101 Sherbrooke East, Montréal, QC H1X 2B2, Canada; sougatairbv20@gmail.com; 2 Institute of Marine & Environmental Technology, University of Maryland Center for Environmental Science701 E. Pratt St., Baltimore, MD 21202, USA; jagus@umces.edu

**Keywords:** dinoflagellate, post-transcriptional control, gene expression, translation factors

## Abstract

Dinoflagellates are unicellular protists that feature a multitude of unusual nuclear features, including large genomes, packaging of DNA without histones, and multiple gene copies organized as tandem gene arrays. Furthermore, all dinoflagellate mRNAs experience trans-splicing with a common 22-nucleotide splice leader (SL) sequence. These features challenge some of the concepts and assumptions about the regulation of gene expression derived from work on model eukaryotes such as yeasts and mammals. Translational control in the dinoflagellates, based on extensive study of circadian bioluminescence and by more recent microarray and transcriptome analyses, is now understood to be a crucial element in regulating gene expression. A picture of the translation machinery of dinoflagellates is emerging from the recent availability of transcriptomes of multiple dinoflagellate species and the first complete genome sequences. The components comprising the translational control toolkit of dinoflagellates are beginning to take shape and are outlined here.

## 1. Introduction

Dinoflagellates are a diverse group of unicellular protists that exhibit a great range of size, form and lifestyle. These organisms are notable for their symbioses with coral, the production of harmful algal blooms (HAB), and circadian bioluminescence. The free-living species are important primary producers in the marine environment, yet toxins released by HAB species can result in massive fish kills, human and marine mammal intoxications, as well as economic losses in fisheries and tourism. Dinoflagellates are also known for their diverse types of plastids; since plastids have been gained and lost multiple times, considerable insight has been gained into the process of endosymbiosis.

Dinoflagellates are members of the alveolata and are a sister group to the parasitic apicomplexans such as *Toxoplasma gondii* and *Plasmodium falciparum* as well as to the ciliates such as *Tetrahymena* and *Paramecium* [1,2,3]. While the major outlines of the alveolate lineages were established with rRNA phylogenies, protein-coding genes have been used to refine the branching patterns within the dinoflagellates. Protein-based phylogenies indicate the heterotroph *Oxyrrhis marina* and the perkinsosoan parasite *Perkinsus marinus* represent early branching members from the dinoflagellate lineage [4]. Within the dinoflagellates, there are two major clades, the syndinians and what have been termed the “core dinoflagellates” (formerly the Dinophyceae) [5,6]. Roughly half the marine dinoflagellates can grow autotrophically (photosynthetic) and are thought to be the most important eukaryotic primary producers after diatoms. The autotrophic dinoflagellates are found mostly in the core dinoflagellate group and can be either free-living, or associated with a broad range of hosts as endosymbionts, although many species are now recognized to be heterotrophic or mixotrophic [7]. The closely related syndinian dinoflagellates are primarily parasitic [8].

The dinoflagellates all contain alveoli, flattened membrane bound compartments of unknown function lying underneath the plasma membrane, that has given rise to the name alveolata. They also share a number of unusual nuclear characteristics including generally high DNA content, numerous chromosomes that remain condensed during interphase, DNA in a liquid crystal structure without observable nucleosomes and DNA in which 5-hydroxymethyluracil replaces some of the thymine [9,10,11]. Based on these features, the term dinokaryon was coined to designate nuclei in which the chromosomes appear fibrillar in appearance and histones are absent [12,13,14]. Core dinoflagellates have often undergone large scale gene duplication which has given rise to tandem gene arrays [15,16,17]. Tandemly-arrayed gene copies have no identifiable promoters in the intergenic spacers [15,18] and have been proposed to be transcribed into polycistronic mRNAs [19], although this has recently been challenged [20] and remains open to debate. Genomic sequence has now provided evidence that the upstream regions of dinoflagellate genes contain a conserved TTTT motif [17], of particular interest since the dinoflagellates have replaced the TATA-box binding protein (TBP) found in other eukaryotes with a TATA-box like protein (TLF) that binds TTTT instead [21]. It was previously thought that dinoflagellates lacked histone proteins [22] and instead used histone-like proteins for DNA organization [23], but recent work has refined this original view. First, transcriptome studies have revealed the presence of expressed genes encoding four core nucleosomal histones, H2A, H2B, H3, and H4 [24,25]. Conservation of these sequences suggests they are used, albeit not globally since protein levels are extremely hard to detect. Second, dinoflagellates express a dinoflagellate/viral nucleoprotein (DVNP) that appears to have displaced major histone functions in dinoflagellates [26]. DVNP is present in the core dinoflagellates, the syndinian *Hematodinium* sp. and *O. marina*, but is not found in *P. marinus* [27]. Since ribosomal protein phylogeny now firmly places *O. marina* as a lineage basal to both the syndinians and the core dinoflagellates, it seems likely that remodeling of the nucleus occurred after divergence of *P. marinus* and prior to the emergence of *O. marina*. Lastly, and subsequently to acquisition of DVNP, members of the core dinoflagellates acquired one of two distinct types of bacterial HU–like proteins, HLP-1 or HLP-2 [27]. Interestingly, the timing of this acquisition coincides with the appearance of the liquid crystalline structure with arched fibrils that are one of the hallmarks of dinoflagellate chromosomes. Presumably as a consequence of these latter substitutions, the protein-DNA ratio in core dinoflagellates is much lower than in syndinians, such as *Hematodinium* spp, which were previously considered more like typical eukaryotes because of their lower chromosome number, smaller genomes and no obvious gene amplification [16,28].

The unique physical features of dinoflagellate chromosomes are likely to affect both transcription and replication. Dinoflagellate DNA is packaged at a protein:DNA ratio of 1:10, unlike the equimolar ratios found in other eukaryotes [29]. Dinoflagellates possess some of the largest nuclear genomes known among eukaryotes (1500–245,000 Mbp), and have been predicted to contain 38,000–87,000 protein-coding genes [30]. Until recently, large genome sizes had deterred whole-genome sequencing. However, a complete and a partial genome sequence have now been reported for *Symbiodinium kawagutii* and *S. minutum* (which so far have the smallest dinoflagellate genomes) [17,31], with roughly 37,000 and 42,000 genes identified, respectively. A large number of genes (up to 68% in *S. kawagutii*) are found in gene families, suggestive of lineage-specific gene expansion by duplication. However, both transcriptomic [20] and genomic [17] analyses have shown there is a paucity of sequence-specific transcription factors in dinoflagellates, consistent with constant, steady transcription of most genes with fewer genes under sequence-specific transcriptional control.

## 2. Post-Transcriptional Regulation of Gene Expression in Dinoflagellates

Dinoflagellates are the most common and dramatic source of bioluminescence in the ocean [32] and have attracted keen scientific interest from the early pioneers in microscopy in the eighteenth century to modern molecular biologists. Although many dinoflagellates species exhibit bioluminescence, *Lingulodinium polyedra* (formerly *Gonyaulax polyedra*) has a brief but intense luminescent flash that is visible to the naked eye and easily measurable [33]. Bioluminescence by *L. polyedra*, along with photosynthesis [34], cell division [35] and diurnal vertical migration [36] are regulated by an endogenous circadian (daily) clock [37]. Indeed, *L. polyedra* has been used for over 60 years as a model system in which to address the biochemical mechanisms underlying the observed rhythms, and the circadian clock has been shown to control the synthesis of the proteins involved in bioluminescence [38].

Studies on the clock-controlled rhythm of bioluminescence in *L. polyedra* began in the 1950s when it was noticed that light production is 40 to 60 times greater at night than during the day [39]. Light emission in dinoflagellates depends on luciferase-catalyzed oxidation of a high-energy luciferin substrate [40], normally sequestered and protected from oxidation by a luciferin binding protein (LBP) [41]. Both LBP and dinoflagellate luciferase show circadian rhythms in activity and abundance corresponding to the bioluminescence rhythm [42,43] and LBP has a measured peak of synthesis at the time when protein levels are increasing fastest in the cell [42]. Despite the rapid synthesis of LBP during the first several hours of the night phase, LBP mRNA remains constant over a 24-h period indicating that transcription is unlikely to regulate protein synthesis rates. There is a high amplitude to the bioluminescence rhythm because LBP is specifically degraded at the end of the night, by an as yet unknown mechanism.

In *L. polyedra*, the luminescence-related proteins are not the only ones to be circadian-controlled at the translational level. Two-dimensional gel analysis of *L. polyedra* extracts, from cells incorporating radiolabeled amino acids in vivo during the day and night phases, showed that many proteins exhibit circadian changes in synthesis rates [44,45]. However, no change in the amounts of protein synthesized by in vitro translation from poly(A)^+^ RNA extracted from cells during day and night phases was observed, indicating that the mRNA itself is present and translatable at all times of the circadian cycle [44]. Consistent with this, analysis of mRNA recruitment by investigation of polysome profiles showed that during the subjective day phase (that corresponding to the day phase in constant conditions) less than half the ribosomes were present as polysomes [46]. In contrast, following the light to dark transition, polysome size increased and more than 90% of the ribosomes were found as polysomes, supporting the existence of translational control through changes in mRNA recruitment.

Other examples of translational control of specific transcripts in dinoflagellates have been reported. The NADP-dependent isocitrate dehydrogenase (NADP-ICDH) in the tricarboxylic acid (TCA) metabolic cycle also exhibits circadian changes of protein abundance and enzyme activity in *L. polyedra*, whereas its mRNA level remains constant throughout the diel cycle [47]. Circadian changes in protein synthesis, but not mRNA levels, have also been reported for glyceraldehyde 3-phosphate dehydrogenase (GAPDH) [48], peridinin-chlorophyll *a*-binding protein (PCP) [49], and superoxide dismutase [50]. In fact, most studies in this species show post-transcriptional control of gene expression. This includes genes typically under transcriptional control in other eukaryotes, such as cell cycle genes. In plants, fungi and metazoans, the transcription of several conserved S-phase genes such as proliferating cell nuclear antigen (PCNA), ribonucleotide reductase 2, replication factor C, and replication protein A is activated at S-phase entry by the E2F transcription factor, ensuring their timely availability for DNA synthesis (reviewed in [51,52]). In contrast, in *Karenia brevis*, transcript levels for these genes are unchanged over the cell cycle [53]. However, their proteins are maximally expressed during S-phase, suggesting their cell-cycle-dependent expression may be achieved at the level of translation. Microarray studies using *Karenia brevis* showed only 4% of the genes queried had transcript levels that changed by more than 1.7-fold in response to nitrogen or phosphorus limitation [54], and only 9.8% of the genes differed between day and night [55]. Among these latter, less than 1% of the genes queried varied both during growth under light dark (LD) cycles and in constant light [55]. Similarly, a microarray analysis of 3500 genes from *Pyrocystis lunula* revealed that fewer than 10% of gene transcripts increased in abundance by 2-fold and only ~1% of gene transcripts increased in abundance by 4-fold in response to treatment with 1 mM sodium nitrite or 0.5 mM paraquat [56]. In the dinoflagellate *P. lunula,* only 3% of the genes on the microarray were found to exhibit changes in transcript abundance (between 2- and 2.5-fold) during a diel cycle [57]. A similar low magnitude transcriptome restructuring is found concurrent with the entry into stationary phase in *K. brevis* [53] and *Alexandrium minutum* [58].

Next-generation sequencing technologies have recently made genome-scale analyses of these organisms possible, and studies exploiting them confirm a paucity of transcriptional control. For example, a massively parallel signature sequencing (MPSS) analysis of the dinoflagellate *Alexandrium tamarense* transcriptome has shown that of a total of 40,029 signatures, only 18, 2, and 12 signatures were found exclusively in the nutrient-replete, nitrogen-depleted, and phosphate-depleted cultures, respectively [59]. Signature sequencing delivers several million 17 nucleotide transcript-derived fragments adjacent to a common restriction site in the cDNA and provides a quantitative overview of the transcriptome. The presence of bacteria had the most significant impact on the transcriptome, although changes were observed with only ~1% of the total number of transcribed genes, and only ~1.3% signatures were transcriptionally regulated under any condition [59]. This is lower than was found for the related species, *A. fundyense*, where MPSS showed that only 10% of the signatures were differentially expressed under nitrate- and phosphate-limited conditions [60]. Similarly, 454 pyrosequencing in the basal dinoflagellate, *O. marina*, revealed only 9 and 21 transcripts to be up- and down-regulated by saline stress, respectively, a total of 30 out of 7398 cDNA contigs [61]. Only 11 of these 30 sequences varied by more than 2-fold. Lastly, RNA-Seq analysis of *L. polyedra* showed that no transcripts varied significantly over the course of a light-dark or a circadian cycle, indicating no genes were regulated by either light or the circadian clock [62]. Overall, transcriptome studies have shown that although dinoflagellates do employ some transcriptional gene regulation, post-transcriptional regulation is a dominant and well-documented feature of the regulation of gene expression in these organisms. The importance of translational control in *L. polyedra* is underscored by the poor agreement between the amount of a protein and the level of its transcript, as measured for 3199 proteins by MS-based sequencing and by RNA-Seq for their transcripts [63].

## 3. Translational Control of Gene Expression in Eukaryotes

Gene expression is a multistep process that involves the transcription, translation and turnover of messenger RNAs and proteins. Throughout the eukaryotic tree of life, gene regulation at the translational level is widespread and significant. However, only a few studies have quantified the entire cascade on a genome-wide scale. The extent of gene regulation at the translational level has been demonstrated during early *Drosophila* embryogenesis on a genome-wide basis by determining ribosomal density and ribosomal occupancy of over 10,000 transcripts during the first ten hours after egg laying [64]. The diversity of the translation profiles suggests that multiple mechanisms are responsible for modulating transcript-specific translation. Furthermore, mRNAs involved in some biological processes are co-regulated at the translational level at certain developmental stages. In mammalian fibroblasts, absolute measurements of mRNA and protein abundance, along with turnover measures by parallel metabolic pulse labeling, have been investigated for more than 5000 genes [65]. Using this genome-scale investigation it was found that the cellular abundance of proteins is predominantly controlled at the level of translation. Translational control offers a rapid and specific response to the environmental and physiological changes, and transient alterations of genome-wide translational states are frequently observed in non-steady states, such as cell differentiation and stress responses (reviewed in [66,67]). Even in steady state situations, transcripts with the highest abundance are usually not those found in the highest levels on ribosomes [68,69].

The degree of translational control in dinoflagellates is anticipated to be greater than in metazoans since so far the transcriptional responses to different stimuli appear modest (reviewed [70,71]) and there are few sequence-specific transcription factors [20]. However, despite a wealth of data pointing to the importance of regulation of the translation in dinoflagellates little is known about underlying mechanisms. Given the significance of translational regulation in dinoflagellates, it is important to first identify the components of their translational machinery and then to determine what regulatory mechanisms are in place. Data mining of the increasing number of dinoflagellate transcriptomes suggests that translation and its control in these organisms may show some important differences with that of model organisms such as yeast, the model plant *Arabidopsis* and mammals.

### 3.1. Eukaryotic Translation

Protein synthesis is an ancient, conserved and complex multicomponent process in which a mRNA template is decoded to form a protein sequence. Of the three fundamental life processes—transcription, translation and DNA replication—translation is the most conserved. Amino acid polymerization occurs on the ribosome, a dynamic nucleoprotein machine that has inherent peptidyl-transferase activity and utilizes aminoacyl-tRNAs and a range of protein factors. The basic mechanism of protein synthesis is similar in all organisms and can be divided into four stages: initiation, elongation, termination and recycling. Although translational modulation can occur at all of these stages, translational kinetics have revealed that the major regulatory step is initiation which serves as a target for a multitude of regulatory cues [72,73].

### 3.2. Initiation of Translation

Translation initiation refers to the formation of translation-competent ribosomes, where the initiator tRNA (Met-tRNA_i_) in the ribosomal P-site is base-paired to the mRNA start codon. Bacterial ribosomes, such as in *E. coli*, bind to the mRNA by sequence complementarity between the 3′ terminus of the 16S ribosomal RNA (rRNA) and a Shine–Dalgarno (SD) sequence in the 5′ end of the mRNA. Only three initiation factors, IF1, IF2, and IF3 are required to locate the start codon (reviewed in [74]), and the mRNAs are predominantly polycistronic (>60%). However, alternatives to the SD sequence-dependent mechanism are quite widespread, as only 50–70% of all bacterial transcripts contain a SD sequence [75]. In the archaea, most mRNAs either have leader sequences lacking SD motifs, or are lacking a 5′-UTR entirely [76]. In some archaeal species, leaderless mRNAs represent over 50% of all mRNAs [77,78,79] and the mechanism of recruitment is poorly understood [76]. Archaea possess a larger number of translation initiation factors than bacteria (reviewed in [80,81,82]). At least six translation initiation factors, which contain up to three subunits, are described for archaea [81]. They include orthologs to the bacterial/eukaryotic factors IF1/eIF1A and IF2/eIF5B, which are thus universally conserved in all three domains of life. Archaea also contain orthologs to eukaryotic factors that are absent in bacteria; aIF1, aIF2, and eIF4A-like (reviewed in [81,82]).

In contrast to the prokaryotes, eukaryotic translation initiation relies on the 5′-cap structure of mRNA. Ribosomes bind to the 5′-end and travel in a 5′–3′ direction to locate the start codon. Furthermore, eukaryotic initiation involves 13 core initiation factors, some of which are large, multimeric complexes (reviewed in [73]). The eukaryotic translational machinery clearly originated in the archaea, or the common ancestor with archaea, but in addition includes eIF3, eIF4E, eIF4B (some eukaryotes), eIF4G, eIF4H, poly(A) binding protein (PABP) and eIF5. With the exception of eIF5, all eukaryotic-specific initiation factors are involved in mRNA recruitment. For the purposes of this review, eIF6 is considered to be a ribosome anti-association factor and has not been included with the initiation factors. Similarly, eIF2C should not be included as a translation factor since it functions in RNA silencing by binding to microRNAs and repressing the translation of mRNAs complementary to them [83]. Conversely, poly(A) binding protein (PABP), also involved in mRNA recruitment, is generally considered to be a translation initiation factor. With the exception of eIF5, all the eukaryotic-specific initiation factors (eIF4E, eIF4G, eIF4B, eIF4H, eIF3, and PABP) play roles in mRNA recruitment.

**Figure 1:**
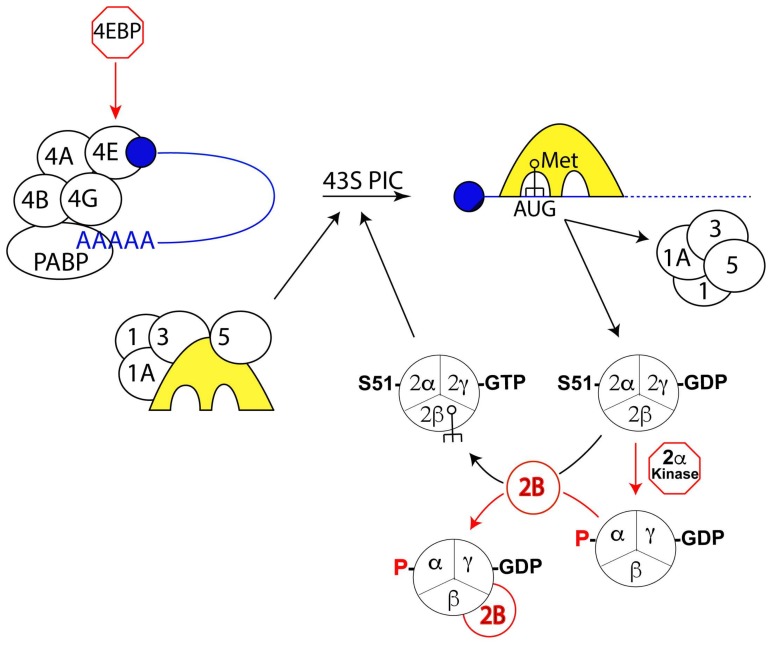
A schematic view of the factors involved in eukaryotic translation initiation. Transcripts (in blue) are present in the cytoplasm as a complex with the poly(A) binding protein (PABP), the cap-binding protein eIF4E, the scaffold protein eIF4G (linking PABP with the cap-binding protein) and two other initiation factors eIF4A and eIF4B. The 43S pre-initiation complex (43S PIC) is formed when the small ribosome subunit (yellow) in a complex with initiation factors eIF1, 1A, 3 and 5 binds the trimeric eIF2, which contains a tRNA charged with methionine (Met-tRNA_i_). The small ribosome subunit moves down the RNA sequence until the anticodon on the Met-tRNA_i_ recognizes and binds the AUG start codon. This recognition allows hydrolysis of the GTP bound to eIF2, causing release of the eIF2 from the PIC along with the eIF1, eIF1A, eIF3 and eIF5. The GTP-bound form of eIF2 is regenerated by the guanine exchange factor eIF2B. Translation can be globally downregulated by phosphorylation of eIF2α, which then tightly binds eIF2B thus impeding recycling of eIF2. Orthologues for a GCN-type eIF2α kinase and the cap-binding regulatory protein 4E-BP have not been identified in the dinoflagellate transcriptome sequence assembly (TSA).

During translation initiation in eukaryotes, the small ribosomal subunit first binds an mRNA at its 5′-end and scans in a 5′ to 3′ direction to locate the initiation codon (reviewed in [84]). At this point, the large subunit joins to form the 80S initiation complex, which is now ready for protein synthesis. This entire process is guided by eukaryotic initiation factors (eIFs) (Figure 1). During eukaryotic cap-dependent translation initiation, a preassembled 43S preinitiation complex (PIC) is formed at the capped 5′-end of the mRNA through the interaction of the cap binding protein eIF4E, the scaffold protein eIF4G, the DExD/H-box helicase eIF4A, and eIF4B, (also eIF4H in mammals) (reviewed in [85,86,87,88]). The PIC itself is made up of the small ribosomal subunit, the initiator tRNA (Met-tRNAi) and the initiation factors eIF1, eIF1A, eIF2, and eIF3. eIF2 is a GTPase, and in its guanosine triphosphate (GTP) bound form facilitates recruitment of the eIF4E-bound RNA to the 43 PIC (reviewed in [85]), while eIF3 recruits the small ribosomal subunit (reviewed in [84,87]). The helicase eIF4A, bound to the scaffold eIF4G, is thought to expose a single-stranded region in mRNA for interaction with the ribosome. In many eukaryotes, eIF4G also harbors a binding site for the poly(A)-binding protein (PABP) that, together with an RNA binding domain in the middle region of mammalian eIF4G, increases the stability of the assembly of eIF4E, eIF4G and eIF4A at the 5′ end of mRNA and promotes circularization of mRNA to improve initiation efficiency in repeated rounds of translation [89].

The 43S preinitiation complex (PIC) scans the mRNA leader in the 5′ to 3′ direction for an AUG codon in a suitable sequence context. During scanning, the 5′ UTR of mRNA is held within a channel on the 40S ribosome formed by the body and the head of the 40S subunit, along with eIF1 and eIF1A. mRNA slides through this channel. Scanning processivity is ensured by keeping the mRNA unstructured and oriented appropriately for “inspection” by the anticodon on the initiator tRNA held in the partial P-site of the 43S PIC [90]. The “inspection” by the initiator tRNA mechanistically involves attempting to establish Watson–Crick base pairing between its anticodon and a nucleotide triplet in the mRNA as it moves through the partial P-site. Base pairing between the anticodon of Met-tRNAi and the AUG in the peptidyl-tRNA (P-) site of the 43S PIC is the initial event in start codon recognition [84,90,91,92]. AUG recognition stops the scanning process and triggers hydrolysis of eIF2-bound GTP to guanosine diphosphate (GDP). Hydrolysis of eIF2-bound GTP is the key step that commits the 43S PIC to initiation at a particular start codon; the two proteins that regulate the G-protein cycle are eIF5, which has both GTPase-activating protein (GAP) and GDP dissociation inhibitor (GDI) functions, and eIF2B, the guanine nucleotide exchange factor (GEF) for eIF2. Upon recognition of the initiation codon, eIF1 is displaced from the P-site, leading to the release of Pi and dissociation of eIF2. The dissociation of eIF1 and eIF2 from the 40S subunit results in a stable 48S mRNA-containing PIC. eIF5B binds to this complex and orients the acceptor stem of Met-tRNAi towards the P-site on the 60S subunit to facilitate subunit joining. eIF5B thus aids release of eIF2-GDP from the 48S PIC which promotes formation of the 80S initiation complex. The 80S complex contains Met-tRNAi base-paired to AUG in the P-site and is ready to begin the elongation phase of protein synthesis [93].

## 4. The Translation Machinery in Dinoflagellates

The translation machinery of dinoflagellates is reviewed from the perspective of its mRNAs, ribosomes and translation initiation factors.

### 4.1. Trans-Splicing of Mrna in Dinoflagellates

In most eukaryotes, the 5′ end of a transcript contains a monomethylated m^7^GTP cap, co-transcriptionally added by a series of enzymatic reactions (reviewed in [94]). However, most if not all of the transcripts in dinoflagellates are post-transcriptionally modified at their 5′ end by *trans*-splicing. The splice leader (SL) RNA [95,96] is thus the source of the cap on the mRNA. SL *trans*-splicing was once considered an anomaly of the kinetoplastids, but subsequent identification of *trans*-splicing in dinoflagellates, euglenozoans and several major invertebrate phyla suggests that this form of RNA processing may represent an evolutionarily important form of regulating gene expression [97,98].

Trans-splicing has been found in all dinoflagellate species examined, including species in the basal lineages such as *Oxyrrhis* and *Amoebophrya* [16,61] and in *P. marinus*, indicating that this processing arose early in dinoflagellate evolution [96,99,100]. Because of the addition of the SL, all mRNA share a common 22 nucleotide sequence (5′-DCCGUAGCCATUUUGGCUCAAG-3′, with D = U, A, or G) at their 5′ end [96]. The identity of the cap structure for dinoflagellate transcripts needs to be determined, but there are more possibilities than the monomethylated m^7^GTP typically found on mRNA or the trimethylguanosine found on small nuclear RNAs. For example, the SL in kinetoplastids contributes four consecutive modified nucleotides referred to as a cap-4 structure [101,102]. Independent of the nature of the cap, it is expected to play an important role in translation initiation. In *Trypanosoma brucei*, a minimal level of mRNA cap ribose methylation has been shown to be essential for viability, and hypermethylation of the cap-4 maximizes translation rates [103]. An unusual structure of the dinoflagellate cap would suggest that the initiation factors recognizing the dinoflagellate cap structure might also show some unique features. 

### 4.2. mRNA Codon Flanking Regions

Analysis of the nucleotide frequency distribution in the flanking positions of the initiation codon, AUG, in eukaryotes has established a consensus sequence that is optimal for recognition by the partial P-site in the small ribosomal subunit [104]. This sequence is termed the Kozak sequence after the investigator who first recognized its importance [104,105]. In vertebrates, this sequence GCCRCC**AUG**G (where R = A or G) has conserved nucleotides at −3 (mostly A) and +4 (G) with respect to the +1 adenine of the start codon AUG (101). This consensus sequence differs between different phylogenetic groups. The consensus sequences of yeast (AAAAAA**AUG**UCU) and plants (AACA**AUG**GC) are similar, but not identical [106,107,108]. Protists such as Amoebozoa (AAAAAA**AUG**RNA), *Plasmodium* (UAAAAAA**AUG**AAN), *Toxoplasma* (GNCAAA**AUG**G) show similar conservation at the −3 and +4 positions. A compilation of transcript flanking sequences from two *Symbiodinium kawagutii* libraries generated RCC**AUG**GCN (R=A, G; N=A, G, C, T) [109], a sequence equivalent to that found in vertebrates and other protists. This sequence is also found in *L. polyedra* superoxide dismutase [110] and in the P-type protein pump in *Symbiodinium* [111].

### 4.3. Ribosomes in Dinoflagellates

At present, little is known about dinoflagellate ribosomes. The *Lingulodinium* transcriptome contains ribosomal proteins comparable to those of higher plant and mammalian genome [17,20,31]. However, when compared with eukaryotic and prokaryotic 5.8S rRNAs, *Prorocentrum micans* 5.8S rRNA revealed several dinoflagellate specific nucleotides. These distinctive nucleotides of 5.8S rRNA are found to be located in specific loops that have the potential to play a structural role in ribosome organization [112]. The size of the small subunit rRNA is similar to what is normally found, but the large subunit rRNA appears slightly smaller than the range of values typically found in eukaryotes [113].

### 4.4. Translation Initiation Factors in Dinoflagellates

**Figure 2:**
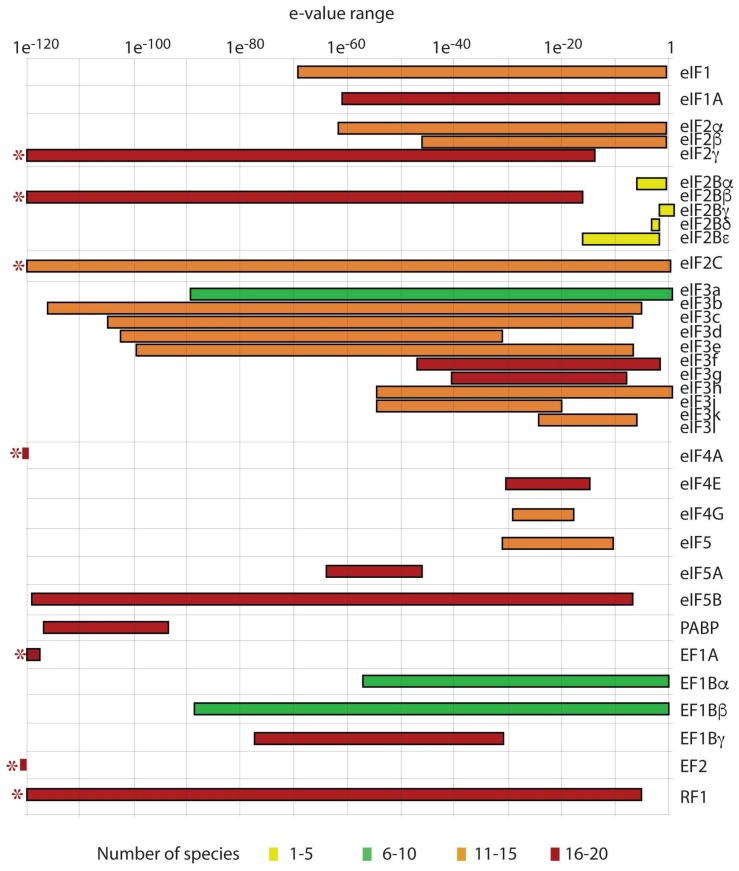
A list of eukaryotic initiation factors from *Arabidopsis thaliana* was used to query the dinoflagellate TSA using tBLASTn. Query sequences are listed at right, and the range of e-values (from e^−120^ to 1) are shown as horizontal red bars; an asterisk at left indicates an e-value of 0. The number of species in which hits were recovered is indicated by the color in the bar. Most of the eIF2B subunits are not found in dinoflagellates. Factors eIF4E. eIF4G and eIF5 are incomplete, suggesting their normal function may be complemented by different proteins in dinoflagellates.

A plethora of transcriptome data from multiple dinoflagellate species combined with the recently available genome sequence from *S. kawagutii* [17] have allowed the identification of translation initiation factors in a wide range of core dinoflagellates. In sharp contrast to the components of the transcription apparatus, the translational machinery in dinoflagellates resembles that of other eukaryotic organisms. This is consistent with the appreciable conservation of translation initiation factors evident for apicomplexans (reviewed in [114]), kinetoplasts (reviewed in [115,116]) and diatoms. Annotation of the *L. polyedra* transcriptome [20] first revealed almost all components of the eukaryotic translational machinery, and these results are confirmed by a more recent survey of the GenBank transcriptome sequence assembly (TSA) using eIF sequences from the vascular plant *Arabidopsis thaliana* [117] (Figure 2). Translational initiation factors not recognized so far include eIF4B and four of the five eIF-2B subunits. eIF4B also appears to be lacking in the genomes of apicomplexa and kinetoplasts, although the sequence is poorly conserved between distant eukaryotes and so may be difficult to recognize. The eIF4G-like sequences found so far seem incomplete, and some of the subunits of eIF3 core complex have not yet been definitively identified.

### 4.5. eIF4E

**Figure 3:**
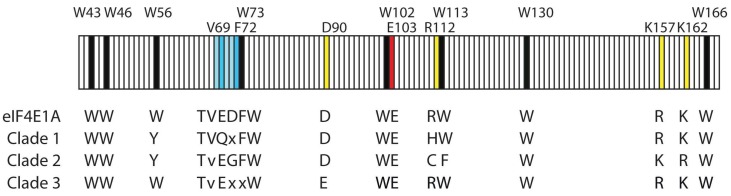
A 130-amino acid conserved core of the mammalian eIF4E1A (subclade1a) illustrating the important binding regions for the m^7^G cap and eIF4G. The eight conserved tryptophan residues (numbered as for the mammalian sequence) are shown in black along with the residues that are found at the corresponding positions in representative sequences from the three dinoflagellate clades. Residues in yellow are important for binding the phosphates of the m^7^G (the aspartate at position 90 coordinates binding by arginine-157), while the eIF4G binding motif (S/TVxxFW) is shown in blue. The small v in the eIF4G binding motif could also be T (for clade 2) or A (for clade 3). The glutamate at position 103 (red) is involved in hydrogen bonding to the m7G. Note that dinoflagellate clade 1 eIF4Es have an insertion of 12–13 amino acids between W73 and W102, as well as an insertion of 7–9 amino acids between W130 and W166. Figure based on [126].

The cap binding protein eIF4E is a central component in the recruitment of mRNA and regulation of translation (reviewed in [85,86,118,119]). The 5′-cap structure is a feature specific to eukaryotic transcripts, so unsurprisingly, eIF4E is an essential translational initiation factor found only in eukaryotes. It has a unique alpha/beta fold that is considered to have no homologs outside the eukaryotes as determined by sequence comparison or structural analyses [120]. The three-dimensional structure of eIF4E bound to a cap analog resembles a cupped hand in which the cap-structure is sandwiched between two conserved tryptophan residues (Trp-56 and Trp-102 of *H. sapiens* eIF4E) [121,122]. A third conserved Trp residue (Trp-166 of *H. sapiens* eIF4E) and an aspartate (Asp-103) also bind the cap-structure (Figure 3). Arg-112, Arg-157 and Lys-162 make direct contacts with the ribose and phosphate moieties. These basic amino acids generate the positive electrostatic potential that partially neutralizes the charge on the two phosphate groups of m^7^-GDP. Lastly, the motif (S/T)VxxFW-73 is a conserved region involved in binding eIF4G and a range of eIF4E interactive proteins [123].

Duplication of the genes encoding eIF4E seems to have taken place very early during eukaryotic evolution and has given rise to a family of proteins that function not only as prototypical initiation factors but also as regulators of mRNA recruitment and localization. Multiple eIF4E family members have been identified in a wide range of organisms including plants, flies, mammals, frogs, birds, nematodes, fish, and various protists (reviewed in [124]). These multiple family members seem to have resulted from a series of duplications of a single early eIF4E gene. The eIF4E family is made up of structurally related proteins within an organism, all containing a conserved core region of 160 to 170 amino acid residues [124]. Not all family members function as prototypical initiation factors, however. The prototypical eIF4E is considered to be eIF4E-1A of mammals, eIF4E and eIF (iso) 4E of plants, and eIF4E of *S. cerevisiae*. eIF4Es from plants, fungi and metazoans can be grouped into one of three classes [124], and different organisms express between one and eight eIF4E isoforms [124,125].

Dinoflagellates also encode multiple eIF4Es that cluster into three distinct clades, although none of these correspond to the previously described eIF4E classes [126]. However, because the cap structure in dinoflagellates is potentially different from that of other eukaryotes, it was anticipated that their eIF4E orthologs could show atypical features. Indeed, recent work has shown that the eIF4E family members of protists are distinct in many ways from those of non-protist eukaryotes [127]. Analysis of the relationship of selected eIF4E-family members from eleven dinoflagellate species shows clear evidence for three distinct clades [127]. Members of each clade differ significantly from each other, but all bear distinctive features of a cap-binding protein. The three clades can be divided into 9 subclasses, six of which were found in all eleven species [126]. There is considerable complexity in this gene family, with divergence seen at critical amino acids (Figure 3). This suggests that the different family members may have different roles.

eIF4Es from clade 1 are represented in all alveolate lineages, and are the only eIF4Es found in apicomplexans, suggesting the authentic translation initiation factor may be in this clade [126]. However, dinoflagellate clade 1 eIF4Es contain extended sequences between W73 and W102, and W130 to W166 compared to eIF4E-2 and -3, a feature not seen in any plant, fungi or metazoan eIF4E family member. The function of the extension is not known, but the different eIF4E-1 sub-clades show marked heterogeneity at this region. Sequence alignments of eIF4E family members from nine core dinoflagellates [126] show that clade 1 and clade 2 eIF4Es have a Tyr substitution at the position equivalent to Trp-56 in mammalian Class I eIF4Es (Figure 3). A positively charged arginine at position 112 has been substituted with a positively charged histidine in clade 1 eIF4Es and with a non-conservative substitution of cysteine in clade 3 eIF4Es. Clade 1 and clade 3 eIF4Es have the conserved positively charged amino acids, equivalent to Arg-157 and Lys-162 of mammalian eIF4E1As, that are involved in binding the pyrophosphate bridge in metazoan eIF4E-1 [121,126]. The Clade 2 eIF4Es have Lys and Arg at positions equivalent to Arg-157 and Lys-162, respectively.

In non-protist eIF4E family members capable of binding eIF4G, the consensus sequence of the recognition motif is S/TVxxFW ending at W73 [121,122], and similar motifs are conserved in eIF4E sequences from core dinoflagellates [126]. However, there are subtle variations between the different eIF4E-1 sub-clades in the consensus sequence of the binding domain. Clade 2 eIF4Es have a tyrosine substitution at W73, and the region can be polar (TVQEFW), as in eIF4E-1a and -1b, acidic (TVEEFW) as in eIF4E-1c, or basic (TVKGFW), as in eIF4E-1d, suggesting different binding partners. The role of these motifs in dinoflagellates is unclear, since the dinoflagellate version of eIF4G lacks an eIF4E binding region (see below). However, it is to be expected that a range of other eIF4E interacting proteins (4E-IPs) may interact with these sites to play roles in mRNA recruitment or its regulation. An increasing number of proteins, generically known as eIF4E-interacting proteins, modulate the eIF4G–eIF4E interaction [128,129]. These include proteins such as maskin, cup, 4E-T, neuroguidin and others [128,129,130,131]. Sequences with similarity to mammalian neuroguidin, which has three eIF4E binding motifs, are found in both *Lingulodinium* and *Symbiodinium* (e-values of e^−15^). In the axons and dendrites of the mammalian nervous system neuroguidin binds to eIF4E-1A and represses translation of cytoplasmic polyadenylation element (CPE) containing mRNAs [132], but it is not known what role, if any, they may play in dinoflagellates. No sequences encoding the regulatory eIF4E binding proteins (4E-BPs) have been identified in dinoflagellates to date. However, 4E-BPs are not consistently found throughout the eukaryotic tree of life. They are absent from plants and nematodes, but are present in some protists such as *Acanthamoeba castellani*, *Dictyostelium discoideum* and *Galucocystis nostochinearum* [123].

It has been suggested that the eIF4E family may contribute to the extensive translational control over gene expression seen in dinoflagellates [126]. Such a role would be greatly supported by differential transcript binding by the eIF4E family members and/or differential interaction with other components of the translational machinery.

### 4.6. eIF4G

In plants and metazoans, recruitment of capped mRNAs requires, in addition to eIF4E, the large scaffold protein eIF4G that serves to coordinate the assembly of the various translation initiation factors and the 40S ribosomal subunit. However, dinoflagellate transcriptomes do not contain sequences that encode a full-length eIF4G. Instead, all dinoflagellate eIF4G transcripts found to date encode only a portion of the mammalian eIF4G equivalent to the MIF4G/HEAT1 domain. This form is expected to allow interactions with eIF4A, eIF3 and RNA. However, the domains at the amino-terminal of eIF4G, responsible for binding PABP and eIF4E, as well as at the carboxyl-terminal region, responsible for binding the eIF4E kinase along with additional eIF4A binding sites, are missing in the dinoflagellate sequences. The binding domains for eIF4E and PABP allow mammalian eIF4G to coordinate independent interactions with mRNA via the cap and poly(A) tail which promotes circularization of the mRNA and a more efficient re-utilization of recruited mRNAs [133]. The dinoflagellate eIF4G is thus unlikely to be able to fulfill these functions, although it must be noted that multiple truncated atypical eIF4Gs have been found in trypanosomatids, and they have been shown to associate with different eIF4Es, eIF4As and PABPs to support translation [116,134,135]. Furthermore, in *S. cerevisiae*, eIF3 and eIF2 are more critical than eIF4G for stable binding of mRNA to native preinitiation complexes, and this appears to require the same RNA-binding domain found in the dinoflagellate eIF4G [88]. In *Leishmania*, eIF4G-3 contains the domain found in the dinoflagellate eIF4G as well as an eIF4E binding domain close to the amino terminal. It is usually assumed that a PABP binding domain is also required to allow the “closed-loop” conformation which is crucial for efficient recruitment of the 43S PIC. However, eliminating the PABP–eIF4G interaction by deletion or mutation of the PABP-binding domain in eIF4G is not lethal in yeast and deletion of the PABP-binding domain has no effect on yeast cell growth provided that the RNA-binding region in the amino terminus of eIF4G1 (RNA1) is intact [136]. eIF3 contains subunits that bind RNA as isolated proteins, such that eIF3 could interact directly with mRNA in the initiation complex [137].

### 4.7. eIF2

All subunits of the translation initiation factor eIF2 are found in dinoflagellate transcriptomes. GTP-bound eIF2 also binds Met-tRNAi in what is termed a ternary complex, and this complex is essential in delivering the Met-tRNAi to the translational machinery during the initiation step of protein synthesis (reviewed in [84,86,138]). The α-subunit of eIF2 functions in a regulatory capacity, as phosphorylation of eIF2α reduces eIF2 activity as well as the overall rate of translation [138,139,140]. In fungi, metazoans, apicomplexans and trypanosomes, phosphorylation of eIF2α has been shown to regulate mRNA recruitment in response to environmental stress, latency and during entry into S-phase [138]. In vertebrates, five eIF2 kinases have been described, each of which is activated by unique stressors (reviewed in [141,142]). Regulation of translation by eIF2α-kinases has been shown to be critical in the life cycle of apicomplexans such as *Toxoplasma gondii* and *P. falciparum* [143,144,145,146,147]. Phosphorylation of eIF2α plays a key role in sustaining parasite viability when they are in their vulnerable extracellular form and when they transition from the proliferating tachyzoites to encysted bradyzoites forms [144,145,146,147,148]. Furthermore, when growing in glutamine-free media, knockouts of a GCN2-like eIF2α-kinase reduce the levels of eIF2α phosphorylation and cause a severe growth defect [149]. GCN2 kinases bind uncharged tRNAs and are thus activated when levels of amino acids are low. Since dinoflagellates are known to respond to a wide range of stressors such as oxidative stress, nitrogen depletion, phosphate limitation, and osmotic stress, it might be expected that phosphorylation of eIF2α would form part of their translational regulatory repertoire. However, only one sequence in the dinoflagellate transcriptome shotgun assembly shows similarity to the *T. gondii* GCN2-like eIF2α-kinase (9% coverage, e-value 9e^−16^) making its identity as an eIF2α-kinase uncertain. Another *T. gondii* eIF2α-kinase, TgIF2K-A, has significant hits with many dinoflagellate species (best blast hit 6% coverage, e-value 1e^−31^). TgIF2K-A has been proposed to regulate response to unfolded proteins in the ER [144].

The inhibitory effects of the eIF2α-kinases on translation are a consequence of reduced recycling of eIF2-GDP to eIF2-GTP by the guanine exchange factor (GEF) eIF2B (reviewed in [138,139]). eIF2B is a decameric protein, with two copies of the regulatory α, β, and δ subunits, and two copies of the catalytic γ and ε subunits. The αβδ regulatory subcomplex contributes to eIF2-GDP binding through interactions with eIF2α, and it is these interactions that are enhanced by phosphorylation of eIF2α [150]. However, of the eIF2B subunits, only eIF2Bβ has a significant presence in the dinoflagellate transcriptome shotgun assembly (Figure 2). Could eIF2Bβ function alone? A recent study in plants has discovered a new role for eIF2Bβ in mediating viral resistance [151], although it is not known if this requires the other eIF2B subunits to function. Dinoflagellate sequences have been found that have weak homology to the ε subunit, which has been shown to be able to act alone in guanine nucleotide exchange [152].

## 5. Translational Regulation by RNA-Binding Proteins

**Figure 4:**
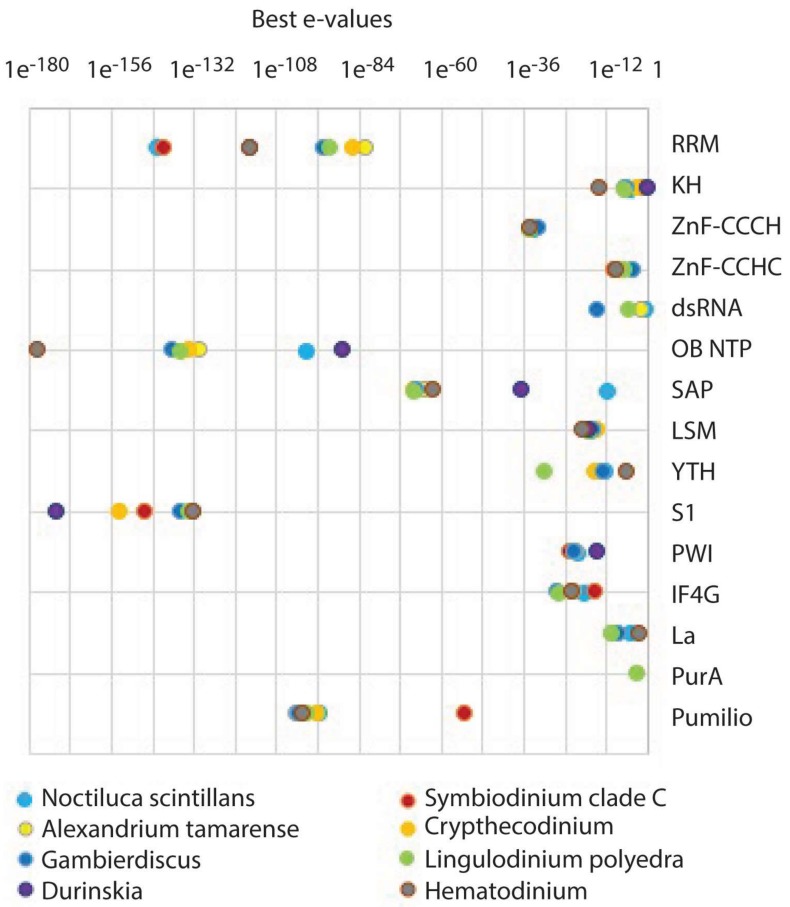
A list of RNA-binding proteins from mammalian cells was used to query the dinoflagellate TSA using tBLASTn, Individual species were queried one-by-one, and the best e-value recorded for each species. Mouse proteins (except where indicated) selected as representatives for the different domains are RRM, polyadenylate-binding protein 1; KH, *Plasmodium* PF3D7_0605100; ZnF-CCCH, splicing factor U2AF 35 kDa subunit; ZnF-CCHC, protein lin-28 homolog; dsRNA, *E. coli* Rnase III; OB NTP-binding, ATP-dependent RNA helicase A; SAP, heterogeneous nuclear ribonucleoprotein U; LSM, LSM14 homolog; YTH, YTH domain-containing family protein; S1, ATP-dependent RNA helicase DHX8; PWI, serine/arginine repetitive matrix protein; eIF4G, eukaryotic translation initiation factor 4 gamma; La, lupus La.

RNA in cells is bound to a number of RNA-binding proteins (RBP) forming ribonucleoprotein complexes that affect splicing, transport to the cytoplasm, translation, stability and subcellular localization [153]. The classical view of RBP is that they bind RNA using specialized RNA binding domains. Thus, one approach to the analysis of RBP in dinoflagellates is to compare sequence homology in the dinoflagellate transcriptome sequencing assembly with a number of known RNA binding domains. A recent publication on mammalian cells has provided a list of 15 RBP containing a variety of different RNA binding domains [154]. When these were used to query eight different dinoflagellate species, four RBPs were found to have the greatest degree of sequence similarity to the dinoflagellates, RRM, the OB NTP fold, S1 and Pumilio (Figure 4).

The poor sequence similarity to members of the other RNA binding protein suggests that the dinoflagellates may have evolved different RNA binding modules. Indeed, new RNA “interactome” studies, in which cross-linking RNA-protein complexes with UV light in vivo is followed by high-throughput proteomics, are providing a quantum shift in how RBP are viewed. Importantly, these proteomic studies, based on function, have revealed that many RBP lack a recognizable RNA binding domain [155]. It is thus possible that many RBPs still await discovery in the dinoflagellates.

The circadian regulation of lbp translation in *L. polyedra* has been correlated with binding of a protein factor to the 3′-UTR of the transcript [156]. Unfortunately, the protein factor was not identified at the time, and subsequent work to repeat the experiments using a different strain of *L. polyedra* have not shown evidence for protein binding by a variety of techniques [157]. 

## 6. Translational Regulation by Small RNAs

RNA interference (RNAi) is a mechanism whereby a few molecules of double stranded RNA introduced into a cell can interfere with gene expression [158]. RNAi is caused by small RNA molecules, termed small interfering RNAs (siRNAs) or microRNAs (miRNAs), and these can regulate translation in both plants and animals [159,160,161,162]. siRNAs, either synthetic or produced naturally, are short double stranded RNA molecules, 20–25 base pairs in length with two nucleotide overhangs on the 3′ ends of each strand. The natural siRNAs are formed by cleavage of longer double-stranded RNAs by an RNase III-like enzyme named Dicer or Dicer-like. The siRNA then bind to an RNA binding protein of the Argonaute/Piwi family, where they are denatured into single-stranded guide RNA. These guide RNAs, usually as part of a larger RNA-induced silencing complex called RISC, target RISC to mRNAs with a sequence complementary to the guide. miRNA differ from siRNAs in that after transcription they form a pre-miRNA molecule which is then processed. Processing involves first the formation of a roughly 60 base hairpin loop structure then, after export to the cytoplasm, cleavage by Dicer. In many eukaryotes, an RNA-dependent RNA polymerase (RdRP) generates double-stranded RNA from single stranded transcripts, thus acting to amplify the RNAi response [163].

The miRNA pathway is widespread but not ubiquitous, as it is not found in phylogenetically diverse organisms such as *Saccharomyces cerevisiae*, *Trypanosoma cruzi*, *Leishmania major* and *Cyanidioschyzon merolae* [164]. The classical pathway is also absent in the apicomplexan *Plasmodium*, a sister group to the dinoflagellates [165], although an abundance of antisense RNAs in the *Plasmodium* transcriptome suggests that these organisms may employ an alternative mechanism [166]. Surprisingly, ciliates (which belong to the same lineage as apicomplexans and dinoflagellates) as well as the heterokonts *Phytophthera*, contain all the components required for miRNA biogenesis and formation of the RISC complex [164]. Other heterokonts, such as the diatoms, lack canonical Dicer/Dicer-like or RdRP genes despite having mi/siRNAs and Argonaute family proteins [167]. This suggests that these organisms must have an alternative to Dicer in order to generate their small RNAs. BLAST searches have identified Dicer-like proteins in *Perkinsus marinus*, a basal lineage in the dinoflagellate clade, as well as in transcriptomes from the core dinoflagellates *Symbiodinium* and *Lingulodinium* [168]. This indicates that Dicer-like enzymes and Argonaute-like proteins are present, although RdRp related proteins have not yet been detected. Searches of the *Lingulodinium* and *Symbiodinium* transcriptomes also contain a protein with RdRp domain as well as several piwi domain-containing proteins (four in *L. polyedra* and two in *Symbiodinium*). The poorly named eIF2C found in dinoflagellates (Figure 2) is a member of the Argonaute family [169]. In mice and humans, this protein binds to miRNAs or siRNAs and represses translation of mRNAs with complementary sequences. It is also involved in transcriptional gene silencing of promoter regions that are complementary to short antisense RNAs bound to the promoter, as well as in the degradation of miRNA-bound mRNA targets [169].

Recent computational approaches identified 21 and 18 predicted small RNAs with miRNA characteristics in *Symbiodinium microadriaticum* and *Alexandrium tamarense,* respectively [170,171]. These predicted miRNAs have both animal (partial complementarity) and plant (perfect complementarity) properties with respect to their targets. In *S. kawagutii*, 102 miRNAs were identified using small RNA sequencing, and 99 of these were also found among the 367 miRNAs predicted from the genome [17]. Of these, the vast majority had partial complementarity to their targets with only one having perfect complementarity. Interestingly, confirmation of miRNA expression was made using Northern blots, and these experiments showed that some of the miRNAs were downregulated by an increase in the temperature from 25 to 35 °C [17]. One of these downregulated miRNAs had heat shock proteins 70 and 90 as predicted targets, thus suggesting a role in regulating gene expression. The fact that miRNAs can in some cases mediate translation rates without affecting mRNA levels is potentially applicable to understanding circadian regulation of translation in *Lingulodinium* where changes in protein synthesis rates are not accompanied by changes in RNA levels. This was tested explicitly using small RNA sequencing to detect miRNAs in this species [168]. However, the results indicate miRNAs do not regulate circadian translation, as out of 9787 sequence reads corresponding to LBP only six were found in an antisense orientation, and none of the 5749 reads corresponding to luciferase were in an antisense orientation. Furthermore, attempts to introduce antisense LBP RNA by biolistic bombardment had no effect on LBP translation.

## 7. Post-Translational Regulation

It is important to note that control over the synthesis of a protein determines the amount but not necessarily the activity of a given protein, as several types of post-translational modifications can intervene to fine tune the levels of active protein. Phosphorylation is the most frequently found post-translational modification (PTM) although many other PTM are known. These include addition of small molecules (acetate, amides, hydroxyls or methyl groups), addition of carbohydrate groups (the process of glycosylation), addition of ubiquitin or small ubiquitin-like modifiers (SUMO), addition of hydrophobic groups such as palmitic acid, myristic acid or prenyl groups), or proteolytic cleavage [172]. Indeed, while protein abundance has long been considered as the most important step in regulating protein function, PTM can affect the structure and function of proteins and can induce effects that can be just as profound [173].

A recent survey of post-translational modifications showed that phosphorylation accounted for roughly two-thirds of the 72,430 experimentally characterized modifications [174]. Furthermore, almost one third of proteins can undergo phosphorylation in eukaryotes [175,176], though for the most part their significance is still unknown. Among those phosphorylated proteins that have been characterized, effects have been found on protein folding, enzyme activity, interactions between proteins, degradation rates and sub-cellular localization [175]. In terms of cellular activities, phosphorylation can be used to regulate signaling, growth, proliferation, differentiation and cell death [177]. Importantly, recent advances in phosphoprotein or phosphopeptide enrichment techniques have made analysis of phosphorylation amenable to analysis by high throughput mass spectroscopy sequencing. This technique requires either genomic or transcriptomic databases, but fortunately, the growing availability of dinoflagellate transcriptome sequence databases is opening the door to studies of proteomics and phosphoproteomics. There are now a sufficiently large number of species represented so that even species without a transcriptome can be analyzed by homology [178]. 

Phosphorylation is also part of the circadian clock mechanism in animals, plants, fungi and cyanobacterial [179,180,181,182]. Furthermore, in *Arabidopsis*, mRNA levels of several kinases and phosphatases are regulated by circadian clock [183,184], and these in turn regulate different rhythmic activities. In *Lingulodinium*, inhibitors of both serine/threonine kinases and phosphatases affect the timing of the bioluminescence rhythm [185,186], supporting a role for phosphorylation in the dinoflagellate clock mechanism. As yet, the full kinase repertoire in dinoflagellates is unknown, although all the kinases found in the transcriptome of *L. polyedra* have been cataloged [187]. It will clearly be of interest to determine the different kinase classes, their abundance and most importantly, their substrates at different times during the circadian cycle. These studies are likely to reveal considerable functional insight. For example, the variation of PCNA abundance with cell cycle stages in *K. brevis* was also accompanied by a shift in electrophoretic mobility consistent with the fact that post-translational modifications of PCNA, such as phosphorylation, are known to control its activity [188]. 2-D polyacrylamide gel electrophoresis (PAGE), phosphoprotein staining and LC-MS/MS after phosphopeptide enrichment were used to assess the phosphoproteome of *L. polyedra* at mid-day and mid-night [189], and the level of 8 among the 45 phosphoproteins varied more than 2-fold between the two times studied. Interestingly, three of these phosphoproteins contained RNA binding domains [189], a finding pertinent to the predominant role of translational regulation in this species.

Lastly, it must also be noted that degradation rates also affect protein abundance in the cell. Unfortunately, protein degradation has not yet been studied in dinoflagellates. In most eukaryotic cells, a ubiquitous protein degradation mechanism involving addition of a small 76 amino acid protein called ubiquitin (Ub) to a target protein controls access to the proteasome, an organelle specialized for protein degradation [190]. Ubiquitination involves a complex of ubiquitin-activating (E1) enzymes, ubiquitin-conjugating (E2) enzymes and E3 ubiquitin ligases, with the choice among many different E2-E3 complexes determining the specificity of the ubiquitination reaction [191]. In both *Symbiodinium* and *Lingulodinium* most of the ubiquitin pathway elements are conserved [24]. BLAST searches have shown that many components to the ubiquitin-proteasome system are found in the transcriptome. In comparison to mammals, however, *Lingulodinium* contains 40% of the E1, E2 and E3 components, similar to what is found in *Arabidopsis*.

While the most well-studied role for ubiquitination lies in mediating protein degradation, the cellular consequences of the PTM depend on where, and how many, ubiquitin moieties are added to a target protein [192]. Target proteins can be mono-ubiquitinated or polyubiquitinated, and in the latter case, ubiquitin groups can be added to different amino acids in the target or to the same amino acid in a series or branched format. Thus, in addition to controlling the amount of protein, ubiquitination can influence protein-protein interactions, in particular by allowing binding to proteins with ubiquitin-binding domains, and this can subsequently regulate the activity and cellular localization of the target protein.

## 8. Conclusions

Dinoflagellates have often found novel ways of carrying out fundamental processes within the cell, making them useful systems in which to study variability in a highly-conserved process such as protein synthesis. Most of the expected players in the initiation of protein synthesis are found in dinoflagellates, at least with respect to their counterparts among the Alveolata. The exceptions are eIF2Bε and γ subunits, eIF4B and eIF4G. In addition, dinoflagellate eIF4G sequences are truncated and atypical, resembling those found in trypanosomatids. It seems likely that variability in mRNA recruitment may explain these differences. Further studies using biochemical approaches should allow functions of the novel eIF4E family members to be uncovered and the apparently “missing” components to be identified in the dinoflagellates, thus providing a unique evolutionary insight into the translational machinery.
